# A Review of Optical-Based Three-Dimensional Reconstruction and Multi-Source Fusion for Plant Phenotyping

**DOI:** 10.3390/s25113401

**Published:** 2025-05-28

**Authors:** Songhang Li, Zepu Cui, Jiahang Yang, Bin Wang

**Affiliations:** College of Information Science and Engineering, Shanxi Agricultural University, Jinzhong 030800, China; lsonghang@163.com (S.L.); cuizepu@126.com (Z.C.); 15136353612@163.com (J.Y.)

**Keywords:** plant 3D reconstruction, active vision techniques, passive vision techniques, precision agriculture, point cloud data

## Abstract

In the context of the booming development of precision agriculture and plant phenotyping, plant 3D reconstruction technology has become a research hotspot, with widespread applications in plant growth monitoring, pest and disease detection, and smart agricultural equipment. Given the complex geometric and textural characteristics of plants, traditional 2D image analysis methods are difficult to meet the modeling requirements, highlighting the growing importance of 3D reconstruction technology. This paper reviews active vision techniques (such as structured light, time-of-flight, and laser scanning methods), passive vision techniques (such as stereo vision and structure from motion), and deep learning-based 3D reconstruction methods (such as NeRF, CNN, and 3DGS). These technologies enhance crop analysis accuracy from multiple perspectives, provide strong support for agricultural production, and significantly promote the development of the field of plant research.

## 1. Introduction

With the continuous advancement of precision agriculture and plant phenotyping research, plant 3D reconstruction technology has become an essential tool in crop growth monitoring, pest and disease detection, and the development of intelligent agricultural equipment [1,2,3]. This review synthesizes research primarily from the last 10 years, emphasizing the significant role of 3D reconstruction in accurately modeling plant morphology, improving the analysis of plant growth status, enabling high-throughput phenotypic trait extraction, and providing a solid scientific foundation for crop variety improvement and precision management [4,5,6]. However, due to the complex geometric morphology and intricate textural features of plants, traditional 2D image analysis methods are insufficient for accurate modeling, which makes the application of 3D reconstruction technology in plant research especially critical [7,8].

Over the past decade, 3D reconstruction techniques have made significant strides. From active vision methods such as structured light, time-of-flight (ToF), and laser scanning, to passive vision techniques like stereo vision and Structure from Motion (SfM), and more recently, advanced deep learning-based methods such as Neural Radiance Fields (NeRF), Convolutional Neural Networks (CNNs), and 3D Gaussian Splatting (3DGS), continuous innovation has opened new frontiers for plant research [9,10,11]. These methods enable the capture of crop morphological features from various angles and at multiple levels, providing comprehensive details ranging from macroscopic plant architecture to microscopic leaf texture and venation. This versatility makes them invaluable tools for precise crop growth analysis, early pest and disease prevention, and optimizing agricultural yield [12,13].

A growing trend in the field is the exploration of multi-source fusion for 3D reconstruction, which combines data from various sensors and integrates 3D models with plant growth physical models. This fusion enhances the accuracy and completeness of the reconstructed models and addresses challenges such as occlusion, wind-induced disturbances, and growth variability [14]. For instance, by combining depth sensors with optical sensors, and integrating these with physiological data, more detailed and reliable plant models can be obtained. Moreover, the application of high-speed imaging systems and event cameras has shown promise in reconstructing dynamic plant scenes, further advancing the real-time capabilities of 3D plant reconstruction technologies.

This review aims to systematically organize the current state and development trends of 3D reconstruction technologies in plant research. We will discuss both active and passive vision techniques as well as deep learning-driven methods, elaborating on their specific applications, advantages, and challenges in plant research. The goal is to provide a thorough overview and offer practical guidance for future research, helping to advance the field and enhance the precision and efficiency of agricultural management. By also considering the potential of multi-source data fusion and its role in improving model accuracy and adaptability, this paper outlines a comprehensive path forward for integrating 3D reconstruction with plant phenotyping in precision agriculture. Table 1 summarizes the technology evolution and application of 3D reconstruction methods.

## 2. Three-Dimensional Reconstruction Technology

Three-dimensional (3D) reconstruction technology refers to the use of specialized equipment or the processing of two-dimensional (2D) image data to create precise 3D models of real-world objects or scenes [15,16]. This technology enables comprehensive capture of the geometric morphology and spatial structure of plants, making it highly valuable in agricultural research, ecological monitoring, and plant biology. Based on data acquisition methods, 3D reconstruction can be categorized into active 3D reconstruction and passive 3D reconstruction [17]. Additionally, according to algorithmic principles, there are deep learning-based 3D reconstruction techniques [18].

### 2.1. Active 3D Reconstruction Techniques

Active 3D reconstruction technique refers to the use of actively emitted signals (e.g., light, laser, infrared, etc.) to measure the distance between the surface of the object and the sensor in the 3D reconstruction process, so as to obtain the 3D information of the object, which plays an important role in the field of plant research [19].

#### 2.1.1. Structured Light Method

The advantage of the structured light method is that it can achieve high-precision 3D information extraction in a large field of view, especially suitable for complex surfaces with rich details [20]. The core principle is based on optical triangulation, where the depth information of an object’s surface is deduced by the known relationship between the relative positions of the light source and the camera, and by measuring the offsets of the light stripes in the camera’s image plane [21]. In practice, a laser transmitter emits a laser beam, which is modulated by the optical system into a striped beam and projected onto the surface of the object [22]. The camera then captures the image of the light pattern on the object’s surface, and subsequently calculates the precise coordinates of the object surface in 3D space according to the calibration equation and the degree of distortion of the light stripes [23].

As it relies on active light source illumination, the structured light method is highly resistant to ambient light interference, can work stably under natural light environments, and has good real-time performance, making it suitable for real-time 3D measurement of dynamic scenes [24].

With its high accuracy and good real-time performance, the structured light method has been widely applied in the field of plant 3D reconstruction, especially in measuring the geometric morphology of plant leaves, the surface texture of fruits, and the overall 3D structure of plants [25]. In agricultural research, the structured light method can be used to monitor plant growth conditions, analyze leaf curl, and evaluate the surface quality of fruits. However, traditional measurement methods, such as 2D image processing or laser scanning, can easily fail to accurately extract the 3D information of an object when the surface features are not prominent (e.g., smooth fruit surfaces or unclear textures) [26]. Chen Xujia et al. proposed a solution to the inability to effectively measure objects due to inconspicuous surface features or occlusion problems in traditional methods. By projecting light stripes of known patterns onto the object’s surface, the deformation of structured light provides obvious depth information, enabling the extraction of 3D morphology from fruit surfaces even in the absence of clear surface features. Furthermore, the relative measurement error of the standard part dimensions through 3D reconstruction is within 3.32%. The deformation index of apples has an *R*^2^ of 0.97, RMSE of 0.755 mm, and mean absolute percentage error (MAPE) of 7.23%. For spherical-like fruits, the *R*^2^ values for volume and maximum diameter are 0.99 and 0.92, with RMSE values of 6.015 cubic centimeters and 1.823 mm, and MAPE values of 1.946% and 1.859%, respectively [27]. This method effectively overcomes the measurement difficulties caused by the lack of clear texture in traditional methods.

#### 2.1.2. Time-of-Flight (TOF)

Time-of-Flight (TOF) is a 3D imaging technique based on measuring the time-of-flight of an optical signal to determine the distance. The technique calculates the distance traveled by a light wave by transmitting a light pulse and accurately measuring the time elapsed from transmission to reception of the reflected light pulse [28]. TOF technology has been widely used in many fields due to its easy operation, fast response speed, and low cost. In particular, in the field of plant 3D reconstruction, TOF can effectively measure a wide range of plant structural information [29].

**Figure 1:**
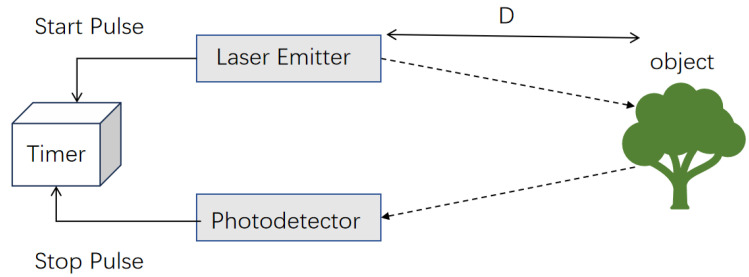
Schematic of a TOF system.

A TOF camera system typically consists of a transmitter (such as a laser or LED light) and a receiver (such as a camera). The transmitter emits short pulses of light waves, which are reflected back after striking the target object, and the receiver captures these reflected light pulses, as shown in Figure 1 [30]. By accurately measuring the round-trip time of the light waves and based on the principle of the constant speed of light, the TOF camera can calculate the distance from the object to the camera using the formula

(1) $$d=\frac{ct}{2},$$

where *d* represents the distance, *c* represents the speed of light, and *t* is the round-trip time of the light wave [31].

In plant science research and agriculture, TOF cameras have been successfully used for 3D modeling of plants, ranging from large-scale trees to small plants [32]. These data are critical for analyzing plant growth adaptations, monitoring pests and diseases, and evaluating crop yields [14]. TOF technology can be applied for accurate measurements of tree height, branch distribution, and fruit count in orchards, assisting agricultural practitioners in optimizing plant cultivation and management plans [33]. Manuel Vázquez-Arellano et al. proposed a 3D reconstruction method for maize plants based on Time-of-Flight (TOF) cameras. They employed the Iterative Closest Point (ICP) algorithm for point cloud registration and used the Random Sample Consensus (RANSAC) algorithm to remove soil points, thus improving the accuracy of the plant point cloud. The method achieves reconstruction by capturing high-resolution 3D images of the maize plants, stitching these point clouds together, and segmenting plant points from soil points. By comparing the positions of the maize seedlings in the generated point cloud with ground-truth measurements, the average deviation of the reconstruction results was 3.4 cm, with a standard deviation of ±1.3 cm [28].

One of the main advantages of TOF technology is that it can perform high-precision measurements under various lighting conditions, especially when dealing with large-scale scenes. However, in environments with highly reflective or extremely dark surfaces, these conditions can affect the effective reception of the light pulses [34].

#### 2.1.3. Laser Scanning Method

The laser scanning method is an active vision 3D reconstruction technique based on laser ranging technology to obtain high-precision 3D point cloud data from the surface of an object [35]. This method uses a laser scanner to emit a laser beam to the target object and measures the time or phase shift of the reflected laser beam returning to the scanner, thus calculating the exact position of each point on the object’s surface [36]. This technology is particularly suitable for accurate mapping of large-scale environments and complex terrains, including forest surveys, farmland analysis, and plant structure studies [37].

A laser scanning system typically consists of a laser transmitter, a receiver, a ranging system, and a computer system [38]. The laser transmitter emits a focused laser beam onto the surface of the target object, and the receiver records the intensity and time information of the reflected laser signal [39]. By precisely measuring these parameters, the system can calculate the distance between the laser and the object and determine its exact position in 3D space. To further enhance measurement accuracy and data reliability, advanced laser scanning systems are typically equipped with Global Navigation Satellite Systems (GNSS) and Inertial Measurement Units (IMUs) to ensure precise positioning of the data over large-scale operations [40].

**Figure 2:**
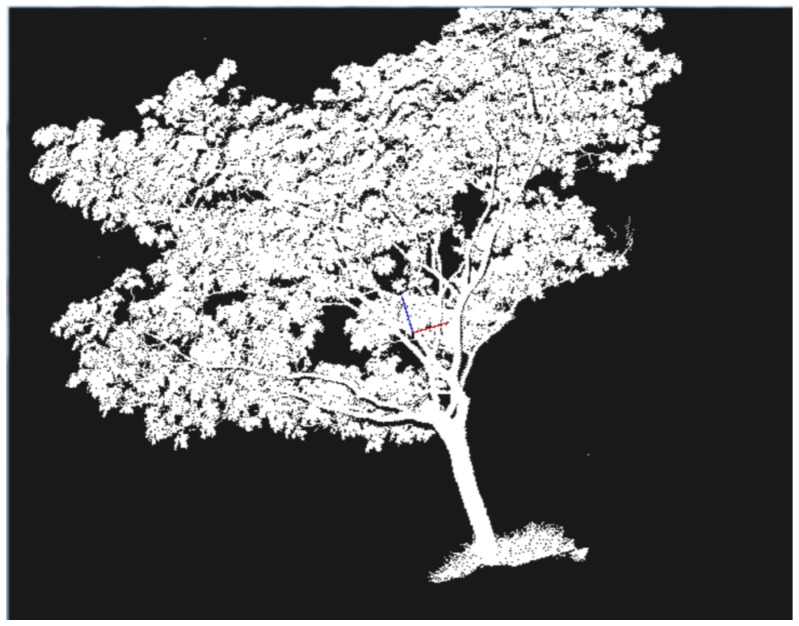
The point cloud generated by the laser scanning method for 3D reconstruction.

In plant science research and precision agriculture, laser scanning technology plays a key role in recording and analyzing plant growth [41]. For instance, laser scanning can accurately measure tree height, branch distribution, and canopy structure. In forest management, these 3D data can be used to estimate timber volume, monitor forest health, and optimize forestry management strategies. Laser scanning is also used for crop growth monitoring. By analyzing the 3D structure of crops, it is possible to assess growth conditions and predict yields [42]. Leonard Freißmuth et al. proposed an online tree reconstruction and forest inventory system based on a mobile robotic platform, which integrates LiDAR-Inertial Odometry (LIO), a graph-based SLAM system, and a Voronoi heuristic clustering algorithm. The researchers developed a “Tree Manager” module that enables real-time tree segmentation, reconstruction, and extraction of tree features such as Diameter at Breast Height (DBH) and trunk curvature. Experiments were conducted in coniferous, broadleaf, and mixed forests in the UK, Switzerland, and Finland to validate the method’s accuracy. The results showed a tree detection recall rate of 98.1%, with an average RMSE of 1.93 cm for DBH measurements, a bias of 0.65 cm, and a standard deviation of 1.81 cm. The average RMSE for trunk curvature was 8.05 cm [43]. The system produces complete results without requiring post-processing and demonstrates real-time reconstruction capability, achieving a good balance between accuracy and efficiency. The point cloud generated by the laser scanning method for 3D reconstruction is shown in Figure 2.

The main advantage of the laser scanning method is its high accuracy and ability to cover large areas. This technology is not affected by lighting conditions, making it effective for use at night or in low-light environments [44]. However, laser scanning can be problematic when encountering transparent or highly reflective surfaces, as these surfaces can interfere with the laser reflection, compromising data accuracy [45]. Additionally, the high cost of laser scanning equipment and its expensive maintenance remain major constraints to its widespread application [46].

#### 2.1.4. Wavelength Selection in Active 3D Reconstruction

In active 3D reconstruction, the choice of emitted wavelength is a critical factor influencing system performance, particularly when capturing plant surfaces with varying reflectance and translucency. Structured light and Time-of-Flight (TOF) systems commonly operate in the visible spectrum (e.g., red, green, blue) or the near-infrared range (NIR). Among these, NIR wavelengths—typically around 850 nm or 940 nm—are widely preferred for plant-related applications due to their lower sensitivity to ambient light fluctuations and improved penetration into plant tissues. These wavelengths correspond well with the high reflectance exhibited by healthy foliage in the NIR region, enabling more reliable surface detection and depth estimation [47]. Moreover, NIR light reduces the influence of plant pigments, enhancing structural contrast even in dense canopies.

For targeted agricultural tasks, avoiding wavelengths with strong absorption by water or pigments can further improve the signal-to-noise ratio. For instance, bypassing chlorophyll absorption bands (such as around 670 nm) helps ensure stronger signal return for depth sensing. In dynamic lighting environments—such as greenhouses or open fields—narrow-band infrared sources combined with optical filtering can greatly enhance system stability and reconstruction quality [48]. Ultimately, tailoring wavelength selection to the spectral properties of plant materials is vital for achieving high-precision reconstruction, particularly when resolving fine structures like leaves, stems, and fruit surfaces.

### 2.2. Passive Three-Dimensional Reconstruction Techniques

Passive 3D reconstruction techniques refer to methods for recovering the 3D structure of an object by analyzing image sequences captured from different viewpoints, without relying on an external emission source, but rather using natural light or existing ambient lighting conditions [49,50]. These techniques offer unique advantages in plant research.

#### 2.2.1. Stereo Vision Method

The stereo vision method is a passive vision 3D reconstruction technique that simulates how the human eye acquires depth information to reconstruct 3D structures. The basic principle involves analyzing two or more images of the same scene captured from different angles and deducing the depth information of each point in the scene by accurately calculating the parallax [51]. The core idea is to obtain the 3D structural information of the object by utilizing the correspondence between images captured from different viewpoints.

**Figure 3:**
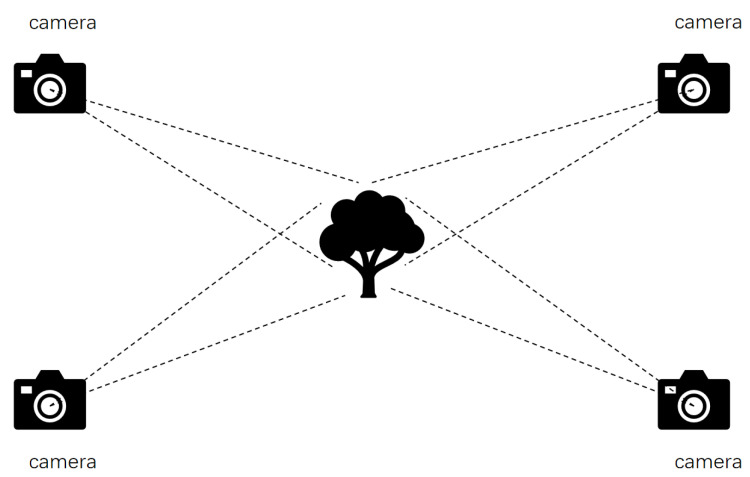
Stereoscopic vision method diagram.

A stereo vision system typically consists of two or more cameras that capture the same scene with a fixed baseline (i.e., the distance between the cameras), as shown in Figure 3. The images captured by each camera provide different views of the scene, and the parallax (the difference in the position of the same scene point in different images) becomes the key to calculating depth information. Using the principle of triangulation, the distance from a point on the object’s surface to the camera can be calculated from the parallax, completing the 3D reconstruction [52].

In plant science research and agricultural applications, stereo vision technology is widely used across various fields such as plant growth monitoring, disease identification, and crop phenotypic analysis [53]. By analyzing plant images taken from different angles, researchers can measure key parameters such as plant growth rates, leaf size, and fruit volume, which are critical for crop breeding and health management [54]. For instance, Sébastien Dandrifosse et al. used the stereo vision method to measure the morphological characteristics of winter wheat, including Leaf Area Index (LAI), Mean Leaf Angle (MLA), Leaf Angle Distribution (LAD), and canopy height. The experiment was conducted in a winter wheat field in Lonzée, Belgium, where two cameras were used to capture overhead images. Image segmentation was performed using both RGB and HSV color spaces, and the Support Vector Machine (SVM) algorithm was employed for soil and plant segmentation to calculate the plant’s morphological characteristics. By adjusting the camera and image resolution during the experiment, the team improved the accuracy of image matching and depth computation, ultimately achieving 99.8% accuracy in segmenting soil, leaves, and spikes. In comparison, the stereo vision method achieved 97.1% accuracy when measuring canopy height, with an RMSE of 0.016 m. The estimation error for the Leaf Area Index (LAI) was RMSE = 0.37 [55].

The primary advantages of the stereo vision method are its relatively simple equipment, low cost, and independence from specific lighting conditions, making it ideal for use in natural environments [56]. Additionally, this technique allows for rapid 3D data collection without physical contact with the plant, thus avoiding any potential physical damage to the plant [57]. However, the stereo vision method also has some limitations. For example, it requires high precision in image alignment, which can lead to errors when dealing with occlusions or scenes with repetitive textures. Moreover, parallax calculation requires advanced algorithms, often involving complex image processing techniques to ensure the accuracy of the data [58].

#### 2.2.2. Structure from Motion (SfM)

Structure from Motion (SfM) is a passive visual 3D reconstruction technique that analyzes a series of images taken from different viewpoints to recover the camera trajectory and reconstruct the 3D structure of a scene. It integrates photogrammetry and computer vision techniques [59], enabling accurate 3D reconstruction without the need for specialized equipment.

One of the key advantages of SfM technology is its low hardware requirements, typically needing only one or more standard cameras to operate [60]. The workflow consists primarily of feature detection, feature matching, motion estimation, triangulation, and bundle adjustment. By tracking and matching common feature points in continuous or semi-continuous image sequences, the SfM algorithm estimates the positions of these feature points in 3D space, as well as the positions and orientations of the cameras during image capture, ultimately generating a dense 3D point cloud model of the scene [61].

In botany and agricultural research, SfM is widely used for 3D modeling across various plant growth stages, from seedlings to maturity [62]. These 3D models not only help researchers analyze plant growth dynamics and morphological changes, but can also be used to assess crop health and predict yields [63]. By analyzing a 3D model of a crop, agricultural experts can more accurately identify early signs of diseases and optimize fertilization and irrigation strategies based on these data, thereby improving crop productivity.

The main advantages of SfM are its flexibility and low cost. As the technology utilizes existing common camera equipment without the need for additional specialized devices, it is particularly well-suited for research environments with limited resources. Additionally, SfM can handle large-scale scenes and generate high-resolution 3D data [64]. However, SfM may experience errors in feature matching when dealing with scenes that have sparse feature points or repetitive textures, which can lead to a decrease in reconstruction accuracy. Furthermore, SfM requires high image quality; factors such as image blur or improper exposure can negatively impact the final 3D reconstruction results. Cao Xin et al. proposed a 3D reconstruction method for field-grown soybean plants that combines Structure from Motion (SfM) with Instant Neural Graphics Primitives (Instant-NGP), referred to as SfM-INGP, aiming to address the high equipment costs and long reconstruction times of traditional field reconstruction approaches. They used consumer-grade smartphones to capture multi-view videos, applied motion detection for adaptive frame extraction to generate image sequences, recovered camera poses using SfM, and performed efficient 3D reconstruction via Instant-NGP. Field experiments were conducted on 56 soybean plants, with 12 plants used for method validation. The results showed that the SfM-INGP method achieved an average reconstruction time of only 2.82 min, representing reductions of 90.7% and 99.4% compared to MVS and NeRF, respectively. The average Peak Signal-to-Noise Ratio (PSNR) reached 24.47 dB, which was 15.4% and 9.3% higher than MVS and NeRF, respectively. The reconstructed Mean Squared Error (MSE) was only 0.15, significantly lower than that of MVS (0.46) and NeRF (0.37) [65].

### 2.3. Deep Learning-Based 3D Reconstruction Technology

Deep learning-based 3D reconstruction techniques are methods that utilize deep neural networks to learn and infer 3D structural information from 2D images.

#### 2.3.1. Neural Radiance Field (NeRF)

Neural Radiance Field (NeRF) is a deep learning-based implicit representation method for 3D scenes, capable of reconstructing highly realistic 3D environments from dense and comprehensively covered multi-view images [66]. NeRF employs a deep neural network to model the color and volume density of each 3D point in the scene and utilizes a differentiable volumetric rendering algorithm to integrate along the camera’s light rays, generating photo-realistic renderings from novel viewpoints. The core innovation of NeRF lies in its high-frequency positional encoding, which enhances detail representation and enables the implicit modeling of complex geometry and appearance [67].

The NeRF model implicitly represents a 3D scene as a five-dimensional radiance field using a multilayer perceptron (MLP). It takes high-frequency encoded 3D coordinates and 2D viewing directions as input, and outputs the volume density and color at each point. Based on known camera parameters, 3D points are hierarchically sampled along light rays, and the final image is synthesized through differentiable volumetric rendering. The MLP is trained by minimizing the mean squared error (MSE) between the rendered and ground truth images, enabling high-fidelity reconstruction of complex geometries and lighting conditions [66].

NeRF has shown great potential in 3D plant reconstruction, particularly in handling complex geometries and fine textures. It has been applied to capture detailed morphological features such as rice panicles and seedling vegetables, providing new tools for agricultural research [68]. This technique is well-suited for automated, accurate plant phenotyping, supporting optimized planting strategies and crop improvement. Zhu Lei et al. proposed a NeRF-based method for 3D modeling and phenotypic extraction of seedling-stage vegetables using smartphone-captured multi-view RGB images. The method reconstructs seedlings like pepper, tomato, and strawberry, followed by point cloud processing—such as line fitting, region growing, and triangulation—to extract traits including plant height, stem diameter, and leaf area. Compared to the SfM-MVS method, NeRF achieved higher reconstruction quality and improved efficiency by an average of 700%. For various plants, point-to-point RMSE ranged from 0.128 to 0.395 cm. For pepper seedlings, *R*^2^ values for plant height and stem diameter were 0.971 and 0.907, with RMSEs of 0.86 cm and 0.017 cm. Leaf area estimation yielded *R*^2^ values of 0.909–0.935 and RMSEs of 0.75–3.22 cm^2^, confirming the method’s accuracy and efficiency in seedling phenotyping [69].

The main advantage of NeRF is its ability to reconstruct highly detailed 3D scenes from limited data, without the need for a prior scene model or complex calibration process [70]. However, the major challenges of NeRF include its high demand for computational resources, especially during the training and rendering phases, and its long processing times, which limit its use in real-time applications. In addition, although NeRF performs well in static scenes, it still faces difficulties when dealing with dynamic and large-scale scenes [66].

#### 2.3.2. 3D Reconstruction Method Based on Convolutional Neural Networks (CNNs)

Convolutional Neural Networks (CNNs), a fundamental deep learning architecture, are widely used in image analysis and have proven effective in 3D reconstruction tasks. By extracting hierarchical features from 2D images through multiple convolutional layers, CNNs can interpret complex visual information and infer 3D structures. As network depth increases, features evolve from low-level edges and textures to high-level representations, enabling accurate reconstruction beyond traditional image classification [71].

In the field of 3D plant reconstruction, CNNs are capable of predicting 3D models of plants from a single image or a small number of images. For example, researchers can use CNNs to analyze the morphology of plant leaves, automatically identifying and quantifying the size, shape, and disease conditions of the leaves [72]. CNNs are also used to monitor plant growth dynamics from time-series images, providing accurate 3D data to support plant growth processes and enabling better decision-making, such as optimizing irrigation and fertilization strategies.

The key advantage of CNNs in 3D reconstruction lies in their high degree of automation and sensitivity to complex visual features, making them well-suited for information-rich images. However, their performance heavily depends on the quantity and quality of training data. They also struggle with occlusion, background noise, and demand high computational resources and training time, which limits practical efficiency [73]. To address structure loss due to occlusion in cotton canopies, Yang Li et al. proposed a CNN-based 3D reconstruction method combining a Cascade Leaf Segmentation and Completion Network (CLSCN) with a Fragmental Leaf Point-cloud Reconstruction Algorithm (FLPRA). The method integrates an Instance Segmentation Network (ISN) and a Generative Adversarial Network (GAN) to reconstruct occluded leaves using RGB-D images and point clouds captured bya RealSense L515 camera (Intel Corporation, Santa Clara, CA, USA). The training set included 2720 images with over 12,000 labeled leaves. Experiments showed the CLSCN network achieved an average mIoU of 87.22% under varying occlusion types, while the GAN module exceeded 94% accuracy in leaf completion with Fréchet Inception Distance (FID) scores below 35. The final reconstructed canopy model reached an accuracy of 82.70%, demonstrating improved completeness and reliability [74].

#### 2.3.3. 3D Gaussian Splatting (3DGS)

3D Gaussian Splatting (3DGS) is an advanced 3D reconstruction method that uses 3D Gaussian distributions to represent and reconstruct scenes. This technology combines the mathematical properties of Gaussian distributions with modern computer graphics and deep learning optimization techniques, enabling efficient and precise reconstruction of complex 3D structures [75].

3DGS represents objects in a 3D scene using a set of 3D Gaussian distributions, each of which has four main attributes: position, shape and size, color, and transparency. The position is represented by the mean of the Gaussian distribution, describing its location in 3D space; shape and size are represented through the covariance matrix; color is represented by spherical harmonic (SH) coefficients, which describe the color and texture of the object’s surface; transparency is controlled by the opacity value (*α*). Through these Gaussian distributions, 3DGS can efficiently describe and render objects in the scene, especially when reconstructing 3D models from 2D images. During the rendering process, initial Gaussian distributions are first derived from images or sparse point cloud data; then, each Gaussian is projected onto the 2D image plane through differentiable rendering, and its color and transparency are calculated. Optimization is performed through back-propagation. Using optimization algorithms such as gradient descent, the parameters of the Gaussian distributions are optimized by comparing the differences between the real and reconstructed images (e.g., L1 loss or SSIM loss), thus achieving high-quality 3D reconstruction [76].

In the field of agriculture, 3D Gaussian Splatting (3DGS) can be applied to crop growth monitoring, plant phenotype analysis, and precision agriculture. By utilizing 3DGS to reconstruct 3D models of crops from 2D images taken from multiple perspectives, researchers can accurately extract various phenotypic features of plants, such as plant height, leaf area, branching distribution, and fruit count [77]. These high-precision 3D models help farmers and researchers better understand the growth state, health condition, and environmental adaptability of crops. Compared with traditional sensors (e.g., LiDAR), 3DGS overcomes the limitations in cost and precision, offering a more economical and efficient solution, especially for large-scale crop monitoring and automated agricultural systems. Lizhi Jiang and others used 3D Gaussian Splatting (3DGS) to successfully reconstruct high-fidelity 3D models of cotton plants and performed detailed 3D cotton boll segmentation. The research team captured images of cotton plants using a smartphone, generated sparse point clouds through photogrammetry, and optimized them into 3DGS models. They developed the Cotton3DGaussians workflow, which performs boll segmentation through multi-view images from four perspectives and removes redundant bolls through cross-view clustering. The study also compared the performance of YOLOv11x and the Segment Anything Model (SAM) in generating 2D instance masks. The results showed that YOLOv11x had an F1 score 5.9% higher than SAM. Using the 3DGS model, the researchers successfully estimated phenotypic features such as boll count, volume, plant height, and canopy width. Compared with LiDAR ground truth data, the model’s mean absolute percentage error (MAPE) for boll count was 9.23%, while the errors for canopy width, plant height, and boll volume were 3.66%, 2.38%, and 8.17%, respectively, demonstrating high accuracy [78].

Table 2 summarizes the above 3D reconstruction methods.

Table 3 summarizes the application examples of the above 3D reconstruction.

## 3. Exploration of Multi-Technology Fusion for 3D Reconstruction of Plants

### 3.1. Multi-Sensor Fusion Techniques

#### 3.1.1. Sensor Types and Functions

Optical Sensors: Including RGB cameras and multispectral cameras. RGB cameras are used to capture the color information of plants, while multispectral cameras record the spectral characteristics of different bands (e.g., near-infrared and mid-infrared), which can support the precise discrimination of various plant parts such as leaves, stems, and fruits [80].

Depth Sensors: For example, depth cameras such as Kinect V2 can acquire 3D point cloud data of plants, providing highly accurate geometric morphological information. These data are particularly crucial for addressing the occlusion problem and can help reconstruct the complete structure of the plant [81].

Thermal Imaging Sensors: These sensors are used to generate images of the thermal distribution of plants, reflecting the temperature status. Such data are valuable for studying the physiological state and health levels of plants [82].

#### 3.1.2. Fusion Methods

Data Fusion: This involves the direct integration of raw data from different sensors at the data layer. For instance, fusing RGB images with depth images to generate multispectral 3D images. This method effectively addresses the occlusion problem, while improving the integrity and accuracy of the data [83].

Feature Fusion: At the feature layer, features are extracted from the data of each sensor and then fused. For example, infrared image features are extracted by Convolutional Neural Networks (CNNs), and visible image features are processed using Transformer networks, followed by their unified integration. This method fully utilizes the complementary advantages of different data modalities and enhances feature expression [84].

Decision Fusion: At the decision layer, the outputs from different sensors are integrated. For example, by matching and integrating the target detection results generated by multiple sensors, more reliable and comprehensive target information is produced. This approach significantly enhances system robustness and reduces false positives and missed detections [85].

#### 3.1.3. Advantages and Challenges of Multi-Sensor Fusion Techniques

(1) Advantages

Enhanced Accuracy and Completeness of 3D Reconstruction: Multi-sensor fusion provides a rich set of information, significantly improving the effectiveness of 3D reconstruction of plants. For instance, fusing RGB images with depth images can effectively solve the occlusion problem and generate complete point cloud models. Additionally, the physiological state information from thermal imaging further enhances the descriptive power of the models [86].

Improved System Robustness: The complementary nature of data from different sensors plays a key role in fusion. By integrating data from LiDAR and cameras, more accurate target detection results can be achieved, reducing the likelihood of missed detections and false positives.

(2) Challenges

Calibration: Data from different sensors must be accurately aligned both temporally and spatially to ensure consistency and accuracy. For example, data from LiDAR and cameras need to be aligned to the same coordinate system [87]. This step is crucial for ensuring the accuracy of subsequent data processing.

Registration: Due to differences in data formats and sampling rates among sensors, appropriate registration is required. For example, data from millimeter-wave radar and LiDAR need to be aligned both temporally and spatially. To improve the efficiency and robustness of registration, a method for coarse registration of single-plant 3D structural point clouds was proposed, which improves registration efficiency by pre-processing point clouds and introducing initial pose constraints [88].

Weight Allocation: During fusion, it is necessary to allocate weights to each sensor’s data based on its reliability. The weighted average method is commonly used to assign weights according to the reliability of each sensor’s data. Recursive estimation algorithms, such as Kalman filtering, can also be applied to fuse uncertain data and dynamically adjust weights for ambiguity and conflicts, thereby improving fusion accuracy [89].

Despite continuous technological advancements, the widespread adoption of 3D reconstruction tools remains limited, particularly in small-scale agricultural settings. Key barriers include the high cost of specialized sensors, substantial computational requirements for running deep learning models, and the lack of accessible training resources for agricultural practitioners. Addressing these challenges will require the development of affordable hardware solutions, the deployment of edge computing technologies, and the design of user-friendly interfaces to lower the threshold for adoption.

### 3.2. Physical Modeling Combined with 3D Reconstruction

#### 3.2.1. Plant Physical Modeling

(1) Growth Model

The growth process of plants is influenced by a variety of factors, including light, temperature, water, and nutrients. The Logistic growth equation is a commonly used mathematical model that effectively controls the growth rate of plants, as well as the allocation of light and resources in each iteration. This equation automatically adjusts the final height of the tree and simulates the growth process of a single tree or multiple trees under the influence of competition [90].

(2) Mechanical Model

The mechanical properties of plants are mainly manifested in their structural stability and deformation behavior. The mass-spring system is a commonly used mechanical model that can effectively simulate the morphology and dynamic changes of plant leaves. For example, motion capture technology can be used to obtain motion data of corn leaves, construct the mass–spring model, and calculate the model parameters using the least squares method to simulate the dynamic behavior of corn leaves [91].

#### 3.2.2. Combined Method and Effect

(1) Combination Method

Data-driven and Physical Model Combination: This method combines 3D real data with plant growth rules, using actual data to guide the process-based modeling of plant growth. For example, Lei Yi et al. [92] proposed a data-driven tree modeling method, assuming that trees have multi-level representations, including main trunks, branches, thin branches, and leaves. By scanning unilateral data using a 3D laser, and combining it with plant growth rules, the missing parts of the branches and trunks are automatically filled using a mechanism based on grammatical rules.

Deep Learning and Physical Modeling Combined: In this approach, deep learning techniques are used to complete incomplete point cloud data, which are then combined with physical models for 3D reconstruction. Haibo Chen et al. [93] proposed a 3D reconstruction method for plants based on a deep camera and deep learning point cloud completion. This method uses deep learning techniques to complement incomplete leaf point cloud data captured by RGB-D cameras under occlusion conditions.

(2) Effectiveness Enhancement

Biological Plausibility: 3D reconstruction methods that integrate physical models significantly improve the biological plausibility of the reconstruction results. For example, by combining data-driven methods with physical modeling, the reconstructed model not only achieves high accuracy (with minimal error compared to real data) but also has higher completeness, aligning with botanical growth patterns [94].

Completeness and Accuracy: The combination of deep learning and physical modeling shows significant advantages in handling occlusion and missing data issues. Experimental results demonstrate that this method has made notable progress in enhancing the completeness of plant point cloud data, especially in the presence of multiple occlusions or high missing data percentages. It also shows more uniform completeness and high effectiveness in leaf area estimation [93].

### 3.3. Dynamic Scene Reconstruction Techniques

#### 3.3.1. Impact of Dynamic Factors and Response Strategies

(1) Wind Impact and Coping Strategies

The impact of wind causes plants to oscillate in space, leading to continuous changes in their form and position. This dynamic change is especially significant at high wind speeds. This requires the reconstruction system to continuously capture and compensate for plant movement to avoid reconstruction errors. To address this challenge, high-speed photography techniques can be employed to reduce motion blur by providing higher temporal resolution data, thus improving reconstruction accuracy in dynamic scenes [95]. Additionally, Event Cameras (ECs), due to their ultra-high temporal accuracy and ability to record light changes in real time without relying on traditional frame rate systems, are particularly suitable for capturing the rapid dynamic changes in plants under the influence of wind and other dynamic factors [96].

(2) Impact of Plant Growth and Motion and Coping Strategies

During plant growth, branches, leaves, and other parts undergo continuous morphological changes due to factors such as light and gravity. These slow dynamic changes can affect the accuracy of 3D reconstruction. To cope with this challenge, plant images and depth data can be collected at regular intervals to capture dynamic changes at different growth stages [97]. In addition, combining data from different time scales and gradually updating the 3D model can reflect the plant’s growth process more accurately, thereby improving the accuracy and biological plausibility of the reconstruction [26].

#### 3.3.2. Applications of High-Speed Cameras, Event Cameras, and Other Equipment

(1) High-Speed Camera Equipment

High-speed camera equipment provides high frame-rate image sequences that can capture the rapid dynamic changes in plants, especially the oscillation of leaves and branches caused by wind. By recording high frame-rate images, these devices reduce motion blur and provide clearer, more accurate image data, significantly improving the accuracy of subsequent 3D reconstruction [98].

(2) Event Cameras

Event Cameras detect brightness changes through independent pixels and can capture real-time information on plant movement, generating a stream of events with temporal information. These streaming data record dynamic changes at high temporal resolution, making it ideal for dynamic scene reconstruction. Event cameras are particularly suited for capturing rapid dynamic changes in plants during wind or growth, excelling in both high accuracy and real-time performance [99].

(3) Algorithm Applications

Deep learning-based algorithms, such as Convolutional Neural Networks (CNNs), can be used to process event stream data, extract key features, and perform 3D reconstruction. For example, the Pix2Vox framework is capable of handling both single- and multi-view 3D reconstruction, generating higher-quality 3D models in dynamic scenes through the use of encoder, decoder, and refiner modules [100].

#### 3.3.3. Time Dimension Information Processing

Capturing dynamic changes is at the core of dynamic scene reconstruction and accurately capturing temporal information is crucial under the influence of plant growth and environmental factors such as wind. Event cameras, by working independently with each pixel and only recording information when brightness changes, effectively avoid the loss of crucial information caused by motion blur in traditional cameras [96]. Their ultra-high temporal resolution enables real-time capture of dynamic plant changes, providing critical temporal information for 3D reconstruction. The EventPS method, through the use of event cameras, efficiently captures light changes and performs real-time surface normal estimation, accurately capturing the dynamic morphology of the plant surface over time, and solving the motion blur and temporal delay issues present in traditional methods [101].

In 3D reconstruction, processing time information is essential, especially in dynamic scenes where plant morphology and structure change over time. Temporal information can serve as the fourth dimension (4D), which, when combined with 3D models, generates dynamic point cloud data. Taking the DymSLAM system as an example, it can reconstruct a 4D point cloud of a dynamic scene through multi-motion segmentation and optimization techniques, handling dynamic changes in moving targets and further improving the accuracy and biological plausibility of the reconstructed model (DymSLAM: 4D Dynamic Scene Reconstruction Based on Geometrical Motion Segmentation). Another deep learning-based technology, NDR (Neural-Dynamic Reconstruction), can generate more accurate dynamic scene geometries by capturing time-varying information and addressing plant growth and wind movement factors in dynamic environments, resulting in 3D models that are closer to reality [79].

## 4. Future Perspectives

### 4.1. Technology Convergence and Innovative Applications

Future developments in 3D reconstruction are expected to move toward deeper integration with emerging technologies such as mixed reality (MR), augmented reality (AR), and virtual simulation platforms. These advances will enable more intuitive visualization and interaction for applications including crop monitoring, spatial analysis, and agricultural decision-making. In parallel, the maturation of unmanned aerial vehicles (UAVs), mobile robotic systems, and ground-based autonomous platforms will facilitate more flexible and real-time acquisition of plant structural data under varying field conditions. The lightweight deployment of reconstruction algorithms on embedded and edge computing devices will further enhance their applicability in environments with limited computational resources.

### 4.2. Intelligence and Automation

Three-dimensional reconstruction is poised to serve as a foundational component of intelligent agricultural systems. When combined with deep learning models, vision transformers, or large pre-trained vision-language architectures such as CLIP and SAM, 3D data can significantly enhance the robustness of feature extraction, semantic segmentation, and instance-level phenotyping across diverse crop species and growth stages [102]. Looking ahead, autonomous field robots equipped with these capabilities may dynamically perform complex tasks such as disease detection, growth monitoring, and adaptive resource application. Moreover, integrating 3D models with AI-driven forecasting tools holds promise for early anomaly detection and more informed agronomic management strategies.

### 4.3. Popularization and Application of Precision Agriculture

As precision agriculture continues to expand, 3D reconstruction will play an increasingly pivotal role in supporting site-specific operations such as precision planting, variable-rate fertilization, and targeted micro-irrigation [4]. These applications demand highly accurate spatial information at both canopy and sub-canopy levels. High-resolution reconstruction techniques—such as Neural Radiance Fields (NeRF) and 3D Gaussian Splatting (3DGS)—enable fine-scale morphological analysis that complements traditional 2D imaging and remote sensing approaches. When linked with physiological measurements or plant growth models, reconstructed 3D representations can support more efficient resource allocation, higher crop yields, and reduced environmental impact.

### 4.4. Data Integration and Decision Support Through Cross-Domain Fusion

Future 3D reconstruction frameworks are likely to be increasingly integrated with big data platforms, cloud computing, and geographic information systems (GISs), enabling scalable storage, processing, and spatiotemporal visualization. By dynamically fusing data from satellites and UAV-based remote sensing systems, multi-resolution and multi-modal crop monitoring pipelines can be established. Additionally, coupling 3D reconstructions with plant growth simulation and agro-ecological models will facilitate 4D modeling—capturing temporal changes in plant structure and physiology. These advancements will enhance agricultural decision support systems by providing actionable insights for land use optimization, stress diagnostics, and yield forecasting [103].

## 5. Summary

Plant 3D reconstruction has become a pivotal technology in supporting precision agriculture and plant phenotyping, with expanding applications in crop growth assessment, intelligent pest and disease detection, and the automation of farm operations. This review presents a comprehensive overview of three principal technological paradigms—active vision, passive vision, and deep learning—alongside widely adopted methods including structured light, time-of-flight (ToF), laser scanning, stereo vision, Structure from Motion (SfM), Neural Radiance Field (NeRF), Convolutional Neural Networks (CNNs), and 3D Gaussian Splatting (3DGS). Each paradigm offers distinct advantages: active vision provides high accuracy and resilience to lighting variability; passive vision stands out for its flexibility and cost efficiency; while deep learning-based approaches demonstrate strong capabilities in modeling complex structures and extracting fine-grained features, positioning them as a growing force in the development of high-fidelity reconstruction technologies.

In real-world agricultural settings, the choice of reconstruction method must be tailored to specific application scenarios. Structured light and ToF systems are particularly effective in controlled environments such as automated greenhouses, where their precision and stability can be fully leveraged. SfM and stereo vision are more suitable for open-field conditions, offering a balance between affordability and adaptability to natural illumination. Deep learning approaches, such as NeRF and 3DGS, are especially well suited for capturing intricate morphological details in static or semi-controlled environments. Meanwhile, LiDAR continues to be the preferred option for large-scale mapping tasks in forestry and orchard management, where dense canopy reconstruction is essential. Key factors—including deployment scale, ambient lighting, sensor mobility, and the required reconstruction resolution—must be carefully considered when selecting a technique.

Beyond the classification of technologies, this paper emphasizes the rising importance of multi-source data fusion. This includes integrating multiple sensors, coupling with plant physiological models, and designing reconstruction pipelines that account for dynamic variables such as wind interference and growth variability. Emerging technologies—such as event-based cameras and high-speed imaging systems—offer new potential for rapid, temporally resolved 3D capture. Looking forward, future research should focus on improving model robustness in complex and variable agricultural environments, enabling real-time processing on embedded platforms, and advancing unified systems that combine 3D modeling with intelligent analysis to support data-driven decision-making in smart agriculture.

## Figures and Tables

**Table 1:** Technology evolution and application of 3D reconstruction methods.

Technology	Development Period	Maturity/Application	Key Features and Applications
Structured Light	Originating in the 1980s, algorithm optimization and system implementation in the 1990s, and gradually maturing after the 2010s	High Precision, Widely Adopted in Controlled Environments	Used for precise plant morphology modeling in controlled environments (e.g., greenhouses).
Laser Scanning	Originating in the 1960s, and with the development of computer technology since the 2000s, it has been widely applied in various domains	Mature in Large-Scale Applications	Applied in large-scale environments (e.g., forestry, orchard), offering high precision and detailed canopy modeling.
ToF (Time-of-Flight)	Started in the 1990s, entered commercial applications in the 2000s, and the development of high-performance compact sensors after 2010 has driven technological advancements	Emerging in Real-Time Applications	Applied in fast dynamic reconstruction for precision agriculture, with strong real-time capabilities.
Stereo Vision	From the development of basic 3D reconstruction using stereo cameras in the 1980s, to the introduction of optimization algorithms in the 1990s, and significant improvements in matching accuracy and application performance in the 2010s	Cost-Effective, Well-Established	Used in open-field crop monitoring under natural light, offering low-cost and flexible solutions.
SfM (Structure from Motion)	Developed starting from the 1980s, optimization algorithms were introduced in the 1990s, and matching accuracy and application performance were significantly improved in the 2010s	Mature, Widely Used in Large-Scale Field Studies	Applied to large-scale plant phenotyping through multi-view image analysis, cost-effective and adaptable.
NeRF (Neural Radiance Fields)	First proposed in 2020, widely applied in multiple fields, and still continuously developing and optimizing	High Fidelity, Emerging in Controlled and Static Environments	Suitable for reconstructing complex plant geometries and fine textures in static or controlled environments with large datasets.
CNN-based Methods	Proposed in the 1980s, rapidly developed in the early 2010s, and matured and widely applied after 2020	Rapidly Growing, High-Adaptability	Highly automated and adaptable for various types of plants, increasingly driving high-accuracy 3D reconstruction in dynamic environments.
3D Gaussian Splatting (3DGS)	First proposed in 2023, widely discussed in academia during 2023-2024, and widely applied across multiple fields since 2024	Emerging, Real-Time Rendering	Effective for generating high-quality 3D scenes in static environments, with relatively fast training speed and real-time rendering capabilities.

**Table 2:** Comparison of 3D reconstruction methods.

Technology Type	Imaging Technology	Principle	Advantages	Disadvantages
Active	Structured Light Method	Encoded structured light	High precision, fast speed, resistant to ambient light interference	Difficult reconstruction for highly reflective or transparent surfaces
	Time-of-Flight (TOF) Method	Reflection light time difference	Cost-effective, simple structure, strong real-time performance	Limited resolution for small targets at close range
	Laser Scanning Method	Laser ranging	High precision, large coverage, non-contact measurement	High equipment cost, expensive maintenance
Passive	Stereo Vision Method	Analyzing image disparity	Low cost, mature technology, suitable for natural light conditions	High camera calibration requirements, accuracy affected by image alignment and synchronization
	Structure from Motion (SfM)	Recovering camera motion and 3D structure from multi-view images	Allows 3D reconstruction from uncalibrated moving devices, strong adaptability	Requires multiple perspective images, high image quality and quantity requirements
Deep Learning	Neural Radiance Fields (NeRF)	Deep learning-based light propagation simulation	Realistic rendering effects, capable of handling complex lighting and details	High computational resource demands, long training and rendering time
	CNN-based Methods	Convolutional neural network extracts features and predicts 3D structures	High automation, strong adaptability	Requires a large amount of annotated data, performance is scene-dependent
	3D Gaussian Splatting (3DGS)	Representing the scene as 3D Gaussian primitives, rendered through differentiable rasterization	High-quality, realistic scenes; fast, real-time rendering; relatively fast training speed	High memory and disk usage; poor compatibility with existing rendering pipelines; supports only static scenes

**Table 3:** Application examples of 3D reconstruction.

Application Scenario	Method	Accuracy/Error	Reference
Shape measurement of spherical fruits (apple, citrus, pear)	Binocular structured light + multi-view point cloud reconstruction	Max diameter RMSE = 1.82 mm, Volume RMSE = 6.01 cm^3^, Deformation index *R*^2^ = 0.97	[27]
Maize plant localization	ToF camera + RANSAC + ICP registration	Position RMSE = 3.4 cm, Std. dev. ±1.3 cm	[28]
Tree trunk diameter reconstruction in forests	Mobile LiDAR + Hough transform + SLAM	DBH RMSE = 1.93 cm	[79]
Wheat canopy structure extraction	Stereo vision + Semi-Global Block Matching (SGBM) + SVM segmentation	Canopy height RMSE ≈ 1.1 cm, Leaf area RMSE = 0.37 cm^2^, Angle error < 1.5°	[55]
Field soybean plant 3D modeling	SfM + Instant-NGP	3D model RMSE = 0.15	[65]
Seedling vegetable phenotyping (e.g., chili, tomato)	NeRF + Point Cloud Library (PCL) analysis	Plant height RMSE = 0.86 cm, Stem diameter RMSE = 0.017 cm, Leaf area RMSE = 0.75∼3.22 cm^2^	[69]
Cotton canopy point cloud reconstruction (with occlusion recovery)	ISN + GAN + FLPRA	Leaf completion RMSE ≈ 0.395 cm, Overall reconstruction accuracy = 82.70%	[74]
Cotton boll 3D reconstruction and trait analysis	Multi-view image + 3D Gaussian Splatting + YOLOv11x	Boll count RMSE ≈ 9.23%, Plant height RMSE ≈ 2.38%, Volume RMSE ≈ 8.17%	[78]
